# Jirō Suzuki (1924–1990)

**DOI:** 10.1007/s00415-025-13393-6

**Published:** 2025-09-19

**Authors:** Mariam M. Yousuf, Hidenori Endo, Jonathan D. Santoro

**Affiliations:** 1 Division of Neurology, Department of Pediatrics, Children’s Hospital Los Angeles, 4650 Sunset Blvd, Mailstop 82, Los Angeles, CA 90027 USA; 2 Department of Neurosurgery, Tohoku University Graduate School of Medicine, Sendai, Miyagi Japan; 3 Department of Neurology, Keck School of Medicine, University of Southern California, Los Angeles, CA USA

**Fig. 1:**
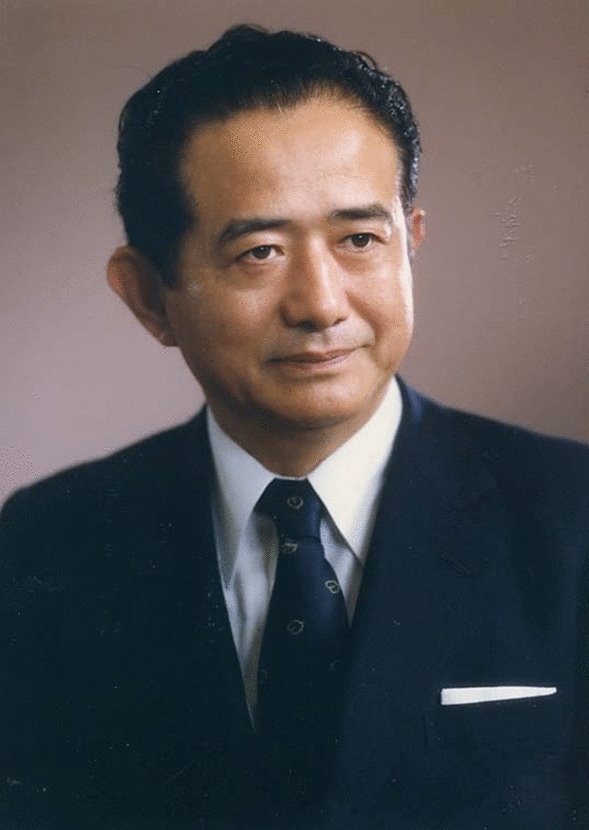
Professor Jirō Suzuki (鈴木 二郎), M.D., Ph.D., courtesy of the Department of Neurosurgery, Tohoku University Graduate School of Medicine

Jirō Suzuki (鈴木 二郎) (Fig. 1) is globally recognized as a pioneer neurosurgeon and physician–scientist whose legacy spans from the discovery of moyamoya disease to advancements in surgical techniques for cerebrovascular disorders. In 1969, Suzuki and his colleague Akira Takaku jointly described moyamoya disease: a rare, progressive cerebrovascular condition primarily affecting children [1]. The disease is characterized by a stenosis of the internal carotid arteries and adjacent branching cerebral vessels. In response, the brain generates a delicate network of fragile compensatory blood vessels which maintain brain perfusion. In cerebral angiography, this network appears as a “hazy puff of smoke,” which inspired the name *moyamoya*, derived from the widely used Japanese term describing this visual phenomenon [1].

Suzuki was born on October 2, 1924, in Sendai City, Miyagi, Japan, into a distinguished, multi-generation family of physicians, representing the seventh generation. During World War II, he served in the Imperial Japanese Navy and rose to the rank of second lieutenant. His time in the Navy would later influence his philosophy of clinical training, leadership, and practice in a patient-first way; he instituted ‘naval exercises’, a 5-min early rule, and rehearsed rounds/case presentations that he carried into the department [2]. After the War, Suzuki entered Tohoku Imperial University School of Medicine in 1946 and earned his M.D. degree in 1950 [2]. Suzuki then trained under professor Shigetsugu Katsura, a pioneer neurosurgeon in Japan, whose mentorship shaped his early career. Suzuki remained in his alma mater as a lecturer and rose through academia to reach associate professor rank in 1964 [2, 3].

In the same year, Suzuki was promoted and thus became the first professor of neurosurgery at Tohoku University, formally establishing the department and launching its dedicated academic journal. In 1967, he became a professor at the newly established Brain Disease Research Institute, and by 1981, he was named its director. Under his leadership, Tohoku University emerged as an international hub for neurosurgical research, particularly in cerebrovascular diseases [3].

One of the hallmarks of Suzuki’s career was his investigation of six unusual cases of cerebral ischemia in children in the late 1950s and early 1960s. These cases involved progressive stenosis of the internal carotid arteries and the formation of a delicate, mesh-like system of tiny vessels, which is most visible in cerebral angiography. Suzuki and Takaku’s seminal 1969 paper in the *Archives of Neurology* formally introduced this condition to the scientific world, describing its imaging features and clinical progression [1].

In a later preface, Suzuki recounted the serendipitous naming of moyamoya disease. Suzuki and Takaku submitted their paper to the *Archives of Neurology* (now *JAMA Neurology*) under the title, “A disease showing abnormal net-like vessels in base of the brain,” with a subtitle of “moyamoya disease.” However, the editor, H. Houston Merritt (1902–1979), inadvertently switched the title and subtitle, bringing moyamoya to the international stage. Suzuki wrote, “For a nicknaming godfather like me, it is a special joy to have this Japanese word enter the honored ranks of medical terminology” [4]. Therefore, what began as a colloquial description of this phenomenon among Japanese physicians eventually became the formal name of a diagnosis recognized and still used worldwide.

Suzuki and Takaku’s characterization of the disease [1] laid the foundation for the modern diagnosis and treatment of pediatric moyamoya-related ischemic stroke [1, 5]. The six angiographic phases, now known as the Suzuki staging system, from carotid fork narrowing to the disappearance of moyamoya vessels with reliance on external carotid and vertebrobasilar collaterals, are still in use to monitor progression and guide surgical planning. The 1983 review in *Stroke* by Suzuki and Namio Kodama crystallized this framework and helped to standardize reporting across medical centers [1, 5].

In addition to characterizing moyamoya disease, Suzuki made significant contributions to neurosurgical techniques for cerebrovascular disorders. One of his early innovations was the trans-Sylvian (transinsular) approach for evacuating hypertensive intracerebral hematomas, developed with Tomohiko Sato in 1976. This method accessed the hematoma via the Sylvian fissure, minimizing cortical disruption. In their 63-case series, the trans-Sylvian group had 1/35 in-hospital deaths, compared with 5/17 after conventional craniotomy and 11/11 in non-operative cases [6].

Suzuki also promoted stereotactic aspiration as a minimally invasive option for putaminal hemorrhage. In a 1989 study of 241 patients, 175 underwent CT-guided aspiration; at 6 months, 51% achieved good-to-excellent outcomes, 7.4% experienced rebleeding, and 6% had died [7].

One of Suzuki’s pivotal contributions was the practice of early surgery for ruptured cerebral aneurysms, especially within 48 h, demonstrating that acute-phase intervention improved survival and functional outcomes after surgery. At a time when delayed elective operations predominated, his groups’ work at Tohoku University reshaped surgical thinking and laid a foundation for early surgery to become widely adopted in aneurysm management worldwide [8].

In addition, Suzuki and his colleagues helped to popularize the bifrontal interhemispheric approach for challenging ACom aneurysms, publishing a 603-case series that serves as a benchmark, particularly for high, posterosuperior projections, where a pterional route often requires gyrus rectus resection. Their technique emphasized bilateral A1/A2 exposure with minimal gyrus rectus manipulation [9]. By 1986, at the age of 63 years, Suzuki had performed over 2,000 cerebral aneurysm operations. His operative volume was exceptionally high for his era and a marker of the disciplined system he created at Sendai [2].

Suzuki retired in 1988 [3]. However, he remained active in the international neurosurgical community. Shortly after retirement, he traveled to the United States to attend the 9th International Symposium on Microsurgery for Cerebral Ischemia, held in Detroit, Michigan. During the return flight to Japan, he suffered a seizure mid-air, prompting an emergency landing in Anchorage, Alaska, where subsequent examinations revealed a glioma in the left basal ganglia. He passed away on June 9, 1990. A memorial service was held on June 25 in Sendai, with more than 1300 attendants paying their respects [10]. His death was deeply felt across neurology and neurosurgery. Besides his research, Suzuki also mentored a generation of leading neurosurgeons who spread his teachings across Japan and internationally, including Korea, China, and South America [10].
